# Short-term supplementation with ω-3 polyunsaturated fatty acids modulates primarily mucolytic species from the gut luminal mucin niche in a human fermentation system

**DOI:** 10.1080/19490976.2022.2120344

**Published:** 2022-09-15

**Authors:** Charlène Roussel, Sara Anunciação Braga Guebara, Pier-Luc Plante, Yves Desjardins, Vincenzo Di Marzo, Cristoforo Silvestri

**Affiliations:** a Institute of Nutrition and Functional Foods (INAF), Faculty of Agriculture and Food Sciences, Laval University, Quebec, QC, Canada; b Centre Nutrition, Santé et Société (NUTRISS), INAF Laval University, Quebec, QC, Canada; c Canada Excellence Research Chair on the Microbiome-Endocannabinoidome Axis in Metabolic Health, CRIUCPQ Laval University, Quebec, QC, Canada; d Faculty of Medicine, Department of Medicine, Laval University, Quebec, QC, Canada

**Keywords:** Ω-3 PUFAs, fish oil, EPA, DHA, gut microbiota, prebiotics, *Akkermansia muciniphila*, mucolytic bacteria, SCFA, M-SHIME® fermentation system

## Abstract

Consumption of omega-3 polyunsaturated fatty acids (ω-3 PUFAs) eicosapentaenoic acid (EPA) and docosahexaenoic acid (DHA) provides multifaceted health benefits. Recent studies suggest that ω-3 PUFAs modulate the gut microbiota by enhancing health-promoting bacteria, such as the mucin specialist *Akkermansia muciniphila*. However, these prebiotic properties have been poorly investigated and direct effects on the gut microbiome have never been explored dynamically across gut regions and niches (lumen *vs*. mucus-associated microbiota). Thus, we studied the effects of 1 week EPA- and DHA-enriched ω-3 fish-oil supplementation on the composition and functionality of the human microbiome in a Mucosal Simulator of the Human Intestinal Microbial Ecosystem (M-SHIME®). Gut microbial communities derived from one individual harvested in two different seasons were tested in duplicate. Luminal and outer mucus-associated microbiota of the ileum, ascending, transverse and descending colons were cultivated over 28 d from fecal inoculates and supplemented with ω-3 PUFAs for the last 7 d. We show that ω-3 PUFA supplementation modulates the microbiota in a gut region- and niche-dependent fashion. The outer mucus-associated microbiota displayed a higher resilience than the luminal mucin habitat to ω-3 PUFAs, with a remarkable blooming of *Akkermansia muciniphila* in opposition to a decrease of *Firmicutes*-mucolytic bacteria. The ω-3 PUFAs also induced a gradual and significant depletion of non-mucolytic *Clostridia* members in luminal habitats. Finally, increased concentrations of the short chain fatty acids (SCFA) propionate in colon regions at the end of the supplementation was associated positively with the bloom of *Akkermansia muciniphila* and members of the *Desulfovibrionia* class.

## Introduction

The multifaceted health properties of ω-3 PUFAs evidenced over the last two decades make them a promising dietary therapeutic approach.^1,2^ In particular, the synergistic effects of the long-chain ω-3 PUFAs, EPA and DHA, found in fish oil, are recognized to provide cardioprotective,^3^ neuroprotective,^4^ anti-depressant,^5^ metaboprotective,^6^ anti-inflammatory^7^ and anticancer properties to the human body.^8^ Although the exact molecular mechanisms underlying their biological effects are still not well understood, recent advances suggested a tight interaction between ω-3 PUFAs EPA/DHA and the gut microbiome in a complex and multidirectional fashion.^9–12^ The gut microbiome, beyond preserving intestinal health, plays critical roles in the maintenance of a healthy state or in the development of a variety of non-communicable gastro-intestinal and extra-intestinal disorders.^13^ It orchestrates interplays with different organs and actively impacts multiple host functions, including metabolism, immunity, circadian rhythmicity, and nutritional responses.^13^ With respect to the latter, gut microorganisms can affect the metabolism and absorption of ω-3 PUFAs;^14^ in turn, ω-3 PUFAs can exert prebiotic properties by enhancing host functions through the modulation of the gut microbiome and their metabolic functions.^9–12^ Many studies have confirmed the prebiotic mechanisms of dietary fibers on health-related traits, but the prebiotic characteristics of ω-3 PUFAs, such as EPA and DHA, remain poorly defined compared to their pharmaconutrient properties.^11^

If one considers the few studies assessing the interplay between ω-3 PUFAs and the gut microbiome, most insights were gained from animal studies and fecal collections of human clinical trials.^9^ Such approaches are respectively limited by clear differences between animal *vs*. human gut physiology, differences in microbial composition between fecal *vs*. intestinal or colonic regions, and the lack of insight into both longitudinal and transversal (e.g., lumen and mucus niches) region-dependent gastrointestinal microbiota signatures.^15^ The mucus layer, at the interface between the digestive luminal content and the host epithelium, remains a neglected niche in most microbiota studies. However, this microenvironment is of particular interest as it constitutes a key feature in the modulation of gut health, providing nutrients and attachment sites for gut microbes.^16^ The O-glycosylated glycoprotein Mucin 2 (MUC2) is the main gel-forming mucin found in the small intestine and colon mucus layer.^17^ It has been shown that ω-3 PUFAs, EPA, and DHA, preserve MUC2 secretion and mucus layer thickness in goblet cell culture assays, in the absence of microbiota.^18^ The mucosa-associated microbiota signature differs from the luminal community by the existence of a biofilm-like organization. In the colon, the mucus is organized in two different layers: the inner membrane-attached and the outer non-attached layer, both displaying distinct composition and accompanied by the floating mucin remaining in the luminal content.^16,17^ It has been suggested that the outer mucus layer and floating luminal mucin nurture mucolytic bacteria dominated by the *Verrucomicrobia* mucus specialist *Akkermansia muciniphila*, as well as species belonging to *Lachnospiraceae* and *Ruminococcaceae*.^16^ Conversely, the inner mucus layer is supposed to be impenetrable to gut bacteria in a healthy state, but some have identified inner mucus layer-attaching bacteria species mostly belonging to *Proteobacteria*.^16^ Importantly, among these few studies it has been shown that ω-3 PUFAs-rich fish oil increases the abundance of *A. muciniphila*, which is known to reduce weight gain and improve glucose metabolism in mouse models or obese humans.^19^ However, the role of ω-3 PUFAs-rich fish oil has never been assessed in the specific niches harboring luminal mucin, inner and outer mucus layer-associated microbiotas, and even less in a dynamic gastrointestinal habitat displaying large longitudinal variation in abiotic and microbiological parameters from the stomach to the distal colon. While it is often recommended to take a food supplement during the winter months to combat “winter blues” or seasonal affective disorders, the prebiotic properties of ω-3 PUFA-rich fish oil have not been explored in gut microbiota collected from opposite seasons of the year.

To fill the gaps, we adapted the well-validated multicompartmental M-SHIME®^20^ to assess the successive human microbiota signatures of the ileum, ascending, transverse, and descending colons. The lumen/mucus interface was also mimicked. Mucin-coated beads were incorporated in the system as a proxy for the physical outer mucus layer, while floating mucin present in the lumen acted as a proxy for the transiting luminal mucin niche. We explored the effects of a 1-week ω-3 fish-oil supplementation enriched in EPA and DHA (compared to a 1-week vehicle) in a host-independent manner, on the composition and functionality of the gut microbiome of one individual in the different gut habitats and niches evaluated over different and opposite seasons (August and February, n = 4).

## Results

### Gut microbiota shows regional habitat- and niche-dependent responses to ω-3 PUFA supplementation

**Figure 1.**
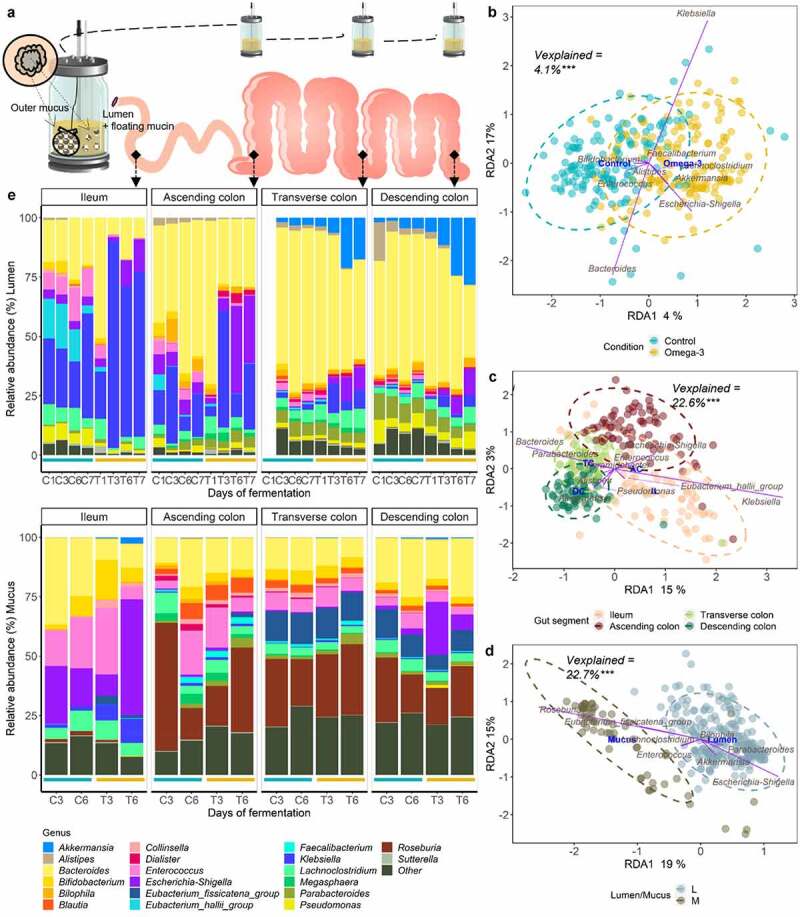
Gut habitats, niches, and ω-3 supplementation as the main explanatory variables to the microbiota community structure at the genus level, in the M-SHIME®. (a) Schematic representation of the different gut habitats and niches recreated in the M-SHIME®. (b-d) Type II scaling triplots obtained using partial distance-based redundancy analysis (db-RDA) of the microbial community composition detected using 16S rRNA gene amplicon sequencing. Treatment condition (b), gut habitat (c) and lumen (l)/mucus (m) niche (d) were set as explanatory variables (in blue) and abundances of genera as response variables (purple arrows). Only the top ten genera were displayed for adequate visibility. Axes are annotated with their contribution to the total variance. “Vexplained” indicates the variability in the gut microbiota composition explained by the variables condition, gut habitat, and lumen/mucus niches. ***indicate the *p* < .001 significance of the observed group separation, as assessed with a Permutational Multivariate Analysis of Variance (PERMANOVA) using distance matrixes. (e) Genus level relative abundance of the luminal (on top) and mucosa-associated (bottom) microbiota composition following a 1-week control (C; blue lines) *versus* 1-week ω-3 treatment (T; Orange lines) at indicated days across the successive gut habitats/niches. For technical reasons (see Methods), mucin beads containing mucosa-associated microbiota were sampled 2 times under each condition. The barplots represent the mean composition of four replicates. The 20 most abundant genera are represented.

The M-SHIME®, harboring luminal (liquid effluents), luminal mucin and outer mucus-associated microbiotas (mucin beads) of the ileum, ascending, transverse, and descending colons was used to investigate the evolution of the microbial community composition over the course of a 1-week supplementation with ω-3 PUFAs, mainly EPA and DHA esterified to triglycerides, compared to a 1-week control (Figure 1a). Two microbiotas from the same donor were tested in duplicate at two distinct seasons (summer and winter, n = 4). The general impact of the ω-3 PUFAs on the microbial community structure was assessed by 16S rRNA gene amplicon sequencing data. The ω-3 PUFA supplementation was an important factor determining the grouping of samples, therefore contributing significantly to the total variation in microbial composition at the genus level (4.1%, F_1,274_ = 11.2, *p* = .001, PERMANOVA), as confirmed by distance-based redundancy analysis (db-DRA) (Figure 1b). The gut habitats and lumen/mucus niches were, however, the dominant explanatory variables (22.6% and 22.7%, respectively, F_3,274_ = 20.7, F_1,276_ = 62.7, *p* = .001, PERMANOVA) (Figure 1c-d). Considering the large gut niche and habitat size effect, the mean changes in microbial community relative abundance under ω-3 PUFA supplementation and control conditions were assessed for each gut niche/habitat separately (Figure 1e). Based on the top 20 most abundant genera, distinct microbiota signatures were observed across habitats. *Gamma-Proteobacteria* such as *Klebsiella* and *Escherichia-Shigella* preferentially colonized the luminal proximal habitats (e.g., ileum, ascending colon), while *Akkermansia* flourished in restricted habitats of the transverse (pH 6.2–6.4) and descending (pH 6.6–6.8) colons (Figure 1e, top). The *Bacteroides* genus increased in distal habitats compared to the proximal one (Figure 1e, top). The *Roseburia* genus efficiently colonized the colon habitats and prevailed in the outer mucus-associated communities with relative abundances reaching on average 25% (Figure 1e, bottom). Individual microbial changes of the 4 samples are provided in Supplementary data Figure S1–2.

ω-3 PUFAs treatment consisted of two capsules, one enriched in EPA and the other in DHA. These capsules each contained 910 mg of one specific fatty acid in triglyceride form out of 960 mg of total ω-3 PUFAs. The total amount of PUFAs in each capsule was 990 mg (Table 1). Free fatty acids EPA and DHA concentrations were tracked across the successive luminal habitats (Table 2). Intriguingly, the dose of EPA/DHA added in the stomach vessel displayed distinct concentrations 2 h after the initial addition, with EPA predominating. Following successive transfer in the ileum, a noticeable increase of EPA and DHA suggests a characteristic metabolism mediated by the ileal microbiota through the breakdown of EPA/DHA triglycerides into free fatty acids. In the colon regions, EPA and DHA-free fatty acids remained at low concentrations, although they gradually accumulated over the 1-week treatment period (Table 2).

**Table 1.** EPA and DHA capsules content. Concentration of different lipids in the capsule based on the products specification’s sheets provided by K.D.-Pharma Bexbach Gmbh.

	EPA enriched capsule	DHA enriched capsule
Fatty acids as triglycerides		
EPA-EE	910 mg/g	<4.4 mg/g
DHA-EE	<4.4 mg/g	910 mg/g
Sum of Omega-3 EE	960 mg/g	960 mg/g
Unsaturated fatty acids	< 1 mg/g	< 1 mg/g
Mono-unsaturated fatty acids	< 1 mg/g	< 1 mg/g
Poly-unsaturated fatty acids	990 mg/g	990 mg/g

**Table 2.** Spatial-temporal fate of EPA/DHA during M-SHIME® fermentation. Concentrations of EPA and DHA as free acids in each gut habitat cumulated over 1 week supplementation is indicated in pmol and gradual amount is illustrated in Orange. EPA and DHA levels are depicted in Orange. T0h refers to the sampling time point before the addition of EPA-DHA in the stomach vessel, deprived of microbiota. Only ileum and colon habitats harbor microbes. Purple arrows indicate the transfer between vessels: stomach and ileum fully empty their contents to follow a given transit time, while colon vessels partially empty their contents to follow a given hydraulic residence time. SI = small intestine.

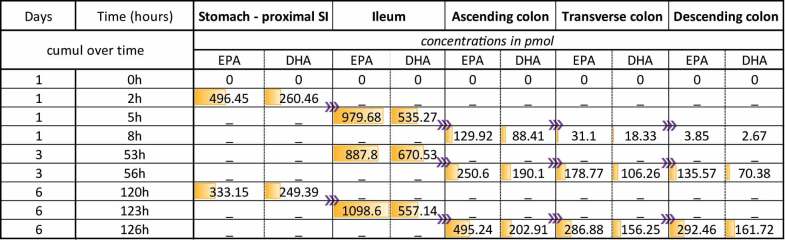

### Outer mucus-inhabiting microbiota display higher robustness than luminal habitats in response to ω-3 PUFA supplementation

**Figure 2.**
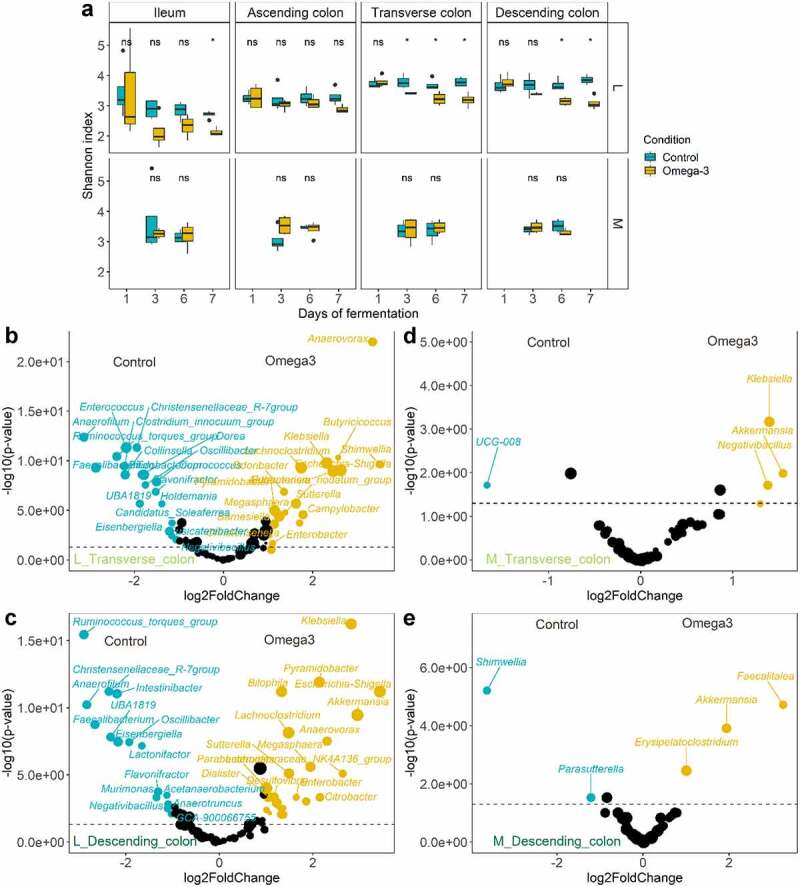
Contrasting response to ω-3 supplementation between luminal/mucus-associated microbiota in the M-SHIME®. (a) Evolution of Shannon diversity index following a 1-week control *versus* 1-week ω-3 supplementation across the successive gut habitats/niches (n = 4 replicates). * represent the *p* < .05 significant differences with control based on Wilcoxon Rank Sum tests with Holm’s correction (α = .05). (b-e) Volcano plots indicating the genera significantly enriched by the ω-3 supplementation in the transverse (b and d) and descending (c and e) gut habitats of the luminal and mucosa-associated (m) niches of the M-SHIME®. A positive log2 fold-change indicates a stimulation of the genus under the ω-3 supplementation period (in Orange) while a negative log2 fold-change indicates a decrease of the genera compared to the control period (in blue), as determined by Deseq2 analysis. Statistical differences between the control and ω-3 PUFA supplementation across gut habitats were determined using a Wald Test. The log transformed adjusted *p*-value is displayed on the y-axis and the α = .05 significance level is indicated by a dashed line.

The ω-3 PUFA supplementation triggered the strongest changes of microbial community structure in distal luminal habitats (Figure 1e, top) and elicited a significant decrease of the microbial diversity at days 6 and 7 compared to the control period (Shannon index, *p* < .05, Wilcoxon Rank Sum tests) (Figure 2a). DESeq-analysis further revealed significant differential relative abundance of many genera between the control and ω-3 PUFA supplementation in luminal and mucus-associated microbiota of the transverse and descending colons (Figure 2b-e), and in luminal ileum and ascending colon (Supplementary data Figure S3). Strong enrichment (> 2 log (base 2) transformed fold change (log2FC), *p* < .05, Wald tests) of *Akkermansia, Escherichia-Shigella* and *Klebsiella* were particularly observed in both luminal transverse and descending colons under ω-3 PUFAs, while *Faecalibacterium, Anaerofilum, Christensenellaceae_R7_group* and *Ruminococcus_torques_group* were depleted (> −2 log2FC, *p* < .05, Wald tests) (Figure 2b-c). On the contrary, ω-3 PUFA supplementation did not alter the Shannon alpha-diversity index of the outer mucosa-associated microbial communities since no significant (ns) change of the Shannon index occurred. This suggests that the microbial communities from the outer mucus layer are resistant to the treatment (Figure 1e, Figure 2a). Indeed, significantly fewer genera were identified as having altered relative abundance within either the transverse (Figure 2d) or descending (Figure 2e) colons. Notably however, as in the lumen niche, *Akkermansia* and *Klebsiella* were both increased in response to ω-3 PUFA supplementation, though the latter only modulated in the transverse colon (*p* < .05, Wald tests) (Figure 2d-e).

### *ω*-3 PUFAs cause a highly significant increase of *Akkermansia muciniphila* together with a decrease of *Firmicutes*-mucolytic bacteria in gut luminal mucin habitats

**Figure 3.**
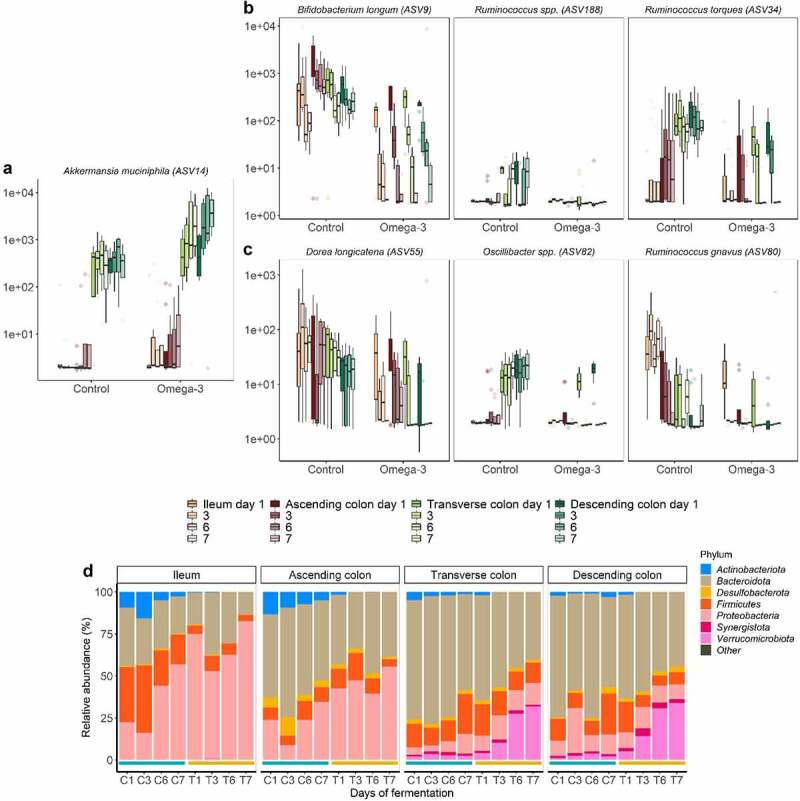
Mucolytic bacteria of luminal floating mucin habitats dynamically shifted following the ω-3 supplementation. (a-c) Selection of mucolytic bacteria that displayed significant differences in ASVs level abundance between control and ω-3 supplementation, as assessed by DESeq2 analysis in the different gut habitats and over time (gradient of colors). Statistical differences between the control and ω-3 PUFA supplementation across gut habitats were determined using a Wald Test. (d) Phylum level relative abundance of the luminal microbiota composition following a 1-week control (C) *versus* 1-week treatment (T) across the successive gut habitats. Control and treatment conditions are demarcated in blue and Orange lines, respectively. The barplots represent the mean composition of four replicates.

**Figure 4.**
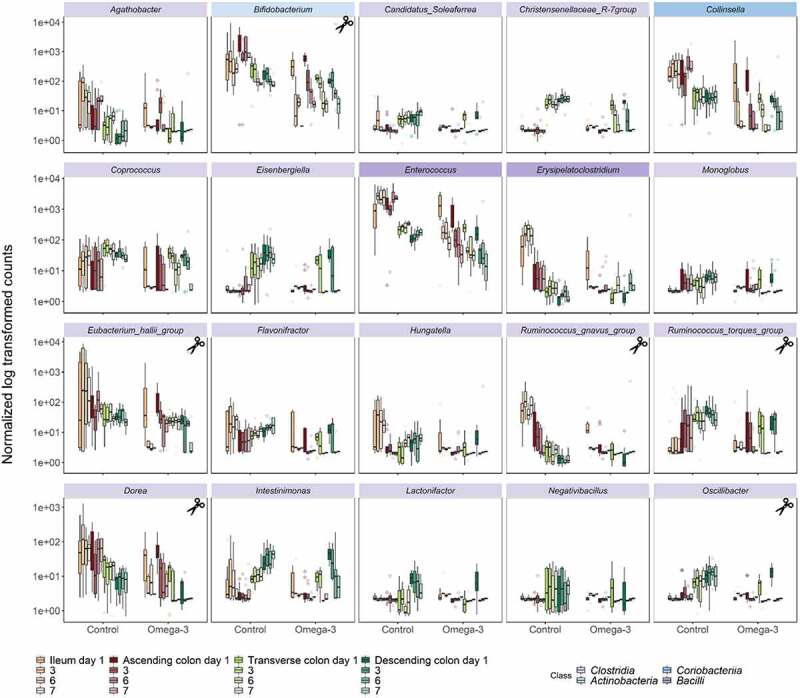
Temporal depletion of genera in luminal floating mucin habitats following the ω-3 supplementation. Bacteria displaying significant differences in genus level abundance between control and ω-3 supplementation, as assessed by DESeq2 analysis in the different gut habitats and over time (gradient of colors). Statistical differences between the control and ω-3 PUFA supplementation across gut habitats were determined using a Wald Test. Colored labels indicate the class of the respective genera. Scissors indicate the genus known for their mucolytic functions.

Mucus, which serves as a fundamental bacterial adhesion niche (for outer mucus-associated microbiota), was present in smaller amounts in the luminal gut habitats (“floating mucin” in luminal microbiota) (Figure 1a). Under ω-3 PUFA supplementation, *Akkermansia muciniphila* (ASV14) displayed significant capacity to flourish in outer mucus associated microbiotas (>2 log2FC, *p* < .05, Wald tests) (Figure 2d-e), with a striking pronounced habitat preference for the floating luminal mucin from the distal habitats (>2 log2FC, *p* < .05, Wald tests), accounting for an approximative 25% of the total relative abundance in transverse and descending colon at days 6 and 7 (Figure 1e). Remarkably, such increase of the keystone *Akkermansia* species following ω-3 PUFA supplementation (Figure 3a) in the floating luminal mucin habitat was associated with a sharp and significant depletion of other mucolytic species (which instead were dominant during the stabilization period or during vehicle supplementation) (Figure 3b-c). These depleted mucolytic species predominantly belong to the *Firmicutes* phylum – i.e., *Ruminococcus torques* (ASV34), *Ruminococcus gnavus* (ASV80), *Ruminococcus spp*. (ASV188), *Oscillibacter spp*. (ASV82), *Dorea longicatena* (ASV55), to the genus *Eubacterium hallii group* (Figure 4), as well as to the *Actinobacteriota* phylum – i.e *Bifidobacterium longum* (ASV9). The dynamics of these decreases displayed a differential temporal pattern, which occurred initially in all gut habitats for *Ruminococcus gnavus* (ASV80), *Ruminococcus spp*. (ASV188), *Oscillibacter spp*. (ASV82), whereas, for the other three species, it occurred only at the end of the supplementation (days 6–7) (Figure 3b-c). Altogether, ω-3 PUFAs caused a strong enrichment of *Verrucomicrobiota* at the expense of *Actinobacteriota* and *Firmicutes* in distal floating luminal mucin habitats (Figure 3d).

### *ω*-3 PUFAs cause a time-dependent, significant, and stable decrease in *Clostridia* abundance

**Figure 5.**
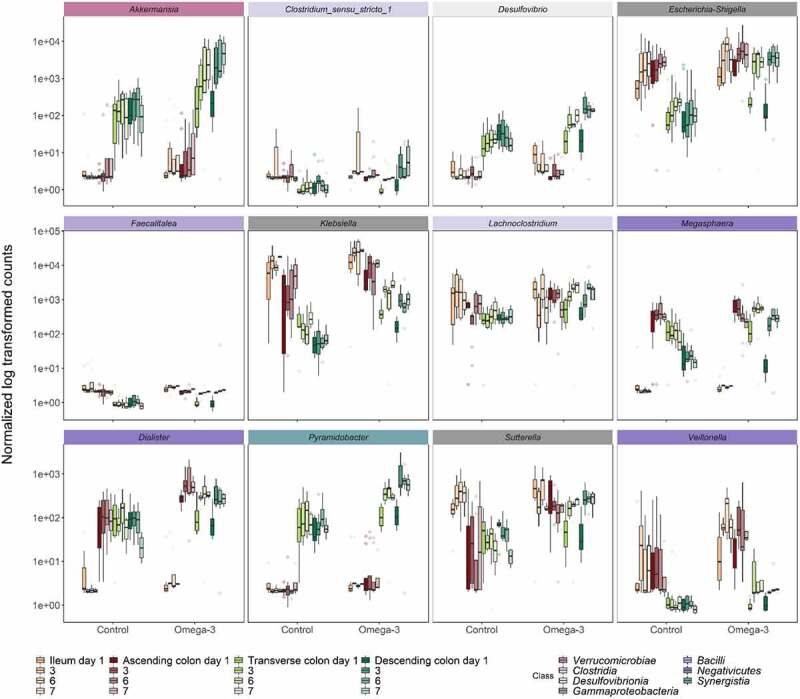
Temporal stimulation of genera in luminal floating mucin habitats following the ω-3 supplementation. Bacteria displaying significant differences in genus level abundance between control and ω-3 supplementation, as assessed by DESeq2 analysis in the different gut habitats and over time (gradient of colors). Statistical differences between the control and ω-3 PUFA supplementation across gut habitats were determined using a wald test. colored labels indicate the class of the respective genera.

In line with the pronounced decline of mucolytic bacteria upon ω-3 PUFA supplementation that predominantly belonged to the *Clostridia* class (*Firmicutes* phylum, *Ruminococcus spp., Oscillibacter spp., Dorea spp*.) (Figure 3b-c, Figure 4), concurrent significant gradual decreases were observed specifically in other non-mucolytic genus members of this class in the gut lumen (Figure 4). *Agathobacter, Coprococcus, Monoglobus, Flavonifractor, Hungatella* and *Intestinimonas* were significantly depleted in all luminal gut habitats over the course of the ω-3 PUFA supplementation. Other *Clostridia* displayed habitat preference for distal segments during the vehicle supplementation, such as *Candidatus_Soleaferrea, Christensenellaceae_R-7_group, Eisenbergiella, Lactonifactor* and *Negativibacillus* and were significantly decreased in response to the ω-3 PUFAs (Figure 4). Strikingly, this time-dependent decrease of *Clostridia* was stable over the course of 1-week supplementation. Independent of the *Clostridia* class, the *Collinsella* genus of class *Coriobacteriia* as well as the *Enterococcus* and *Erysipelatoclostrium* genera of class *Bacilli* were also decreased. On the contrary, genera belonging from *Gamma-Proteobacteria* (*Escherichia-Shigella, Klebsiella, Sutterella*) and *Negativicutes* (*Megasphaera, Dialister, Veillonella*) classes were stimulated under ω-3 PUFA supplementation (Figure 5).

Finally, distinct microbiota compositions in summer and winter exhibited similar responses to ω-3 PUFAs. Indeed, each microbiota showed a unique and seasonal profile of microbial genera abundance at the start of the experiment (fresh fecal inoculation in the summer *vs*. winter from the same donor) (Supplementary data Figure S1–2), while more similar microbial community profiles were obtained in response to the ω-3 PUFA supplementation (Supplementary data Figure S4).

### *ω*-3 PUFAs modulate SCFA profiles in a gut habitat-dependent fashion

**Figure 6.**
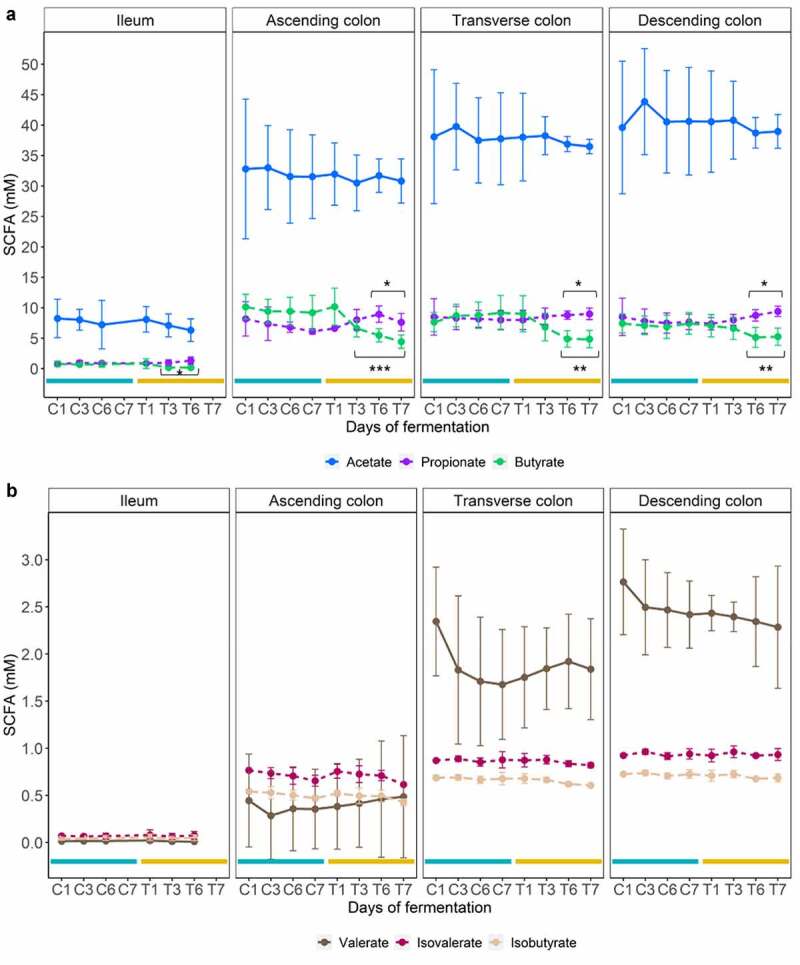
SCFA modulation under ω-3 supplementation. Mean concentrations of major (a) and minor (b) SCFAs in the 4 replicates ± SD across each luminal gut habitat in control (C; blue lines) and treated (T; Orange lines) at different days. Statistically significant differences between control period and ω-3 supplementation are denoted for *p* < .05 (*), *p* < .01 (**), and *p* < .001 (***) as determined by Pairwise Wilcoxon rank sum tests with holm correction.

Short chain fatty acids (SCFA), which are physiologically relevant bacterial fermentation by-products, were measured to study how exposure to ω-3 PUFAs affects general microbial metabolic activity in the different luminal gut habitats (Figure 6). Under control conditions, a gradual increase of the overall SCFA concentrations occurred from the proximal (e.g., ileum) to the distal (e.g., descending colon) gut habitat (Figure 6a). This is indicative of the innate differences in bacterial metabolism with the small and large intestine. Total SCFA concentrations in the ileum remained below 15 mM and acetate contributed essentially to this production, while the colon habitats followed a common ratio of 3:1:1 for acetate, propionate, and butyrate (Figure 6a). Following the ω-3 PUFA supplementation, a significant shift in propionate and butyrate ratio occurred at the end of the intervention predominantly in the colon habitats compared to the control at days 6 and 7 (*p* < .05, Wilcoxon Rank Sum tests). This resulted from, a gradual and significant increase of the propionate concentration (*p* < .01, Wilcoxon Rank Sum tests) at the expense of butyrate (*p* < .05, Wilcoxon Rank Sum tests), which, in contrast, decreased. No significant change in non-dominant SCFAs (e.g., branched chain fatty acids and valerate) was found under ω-3 PUFA supplementation (Figure 6b).

**Figure 7.**
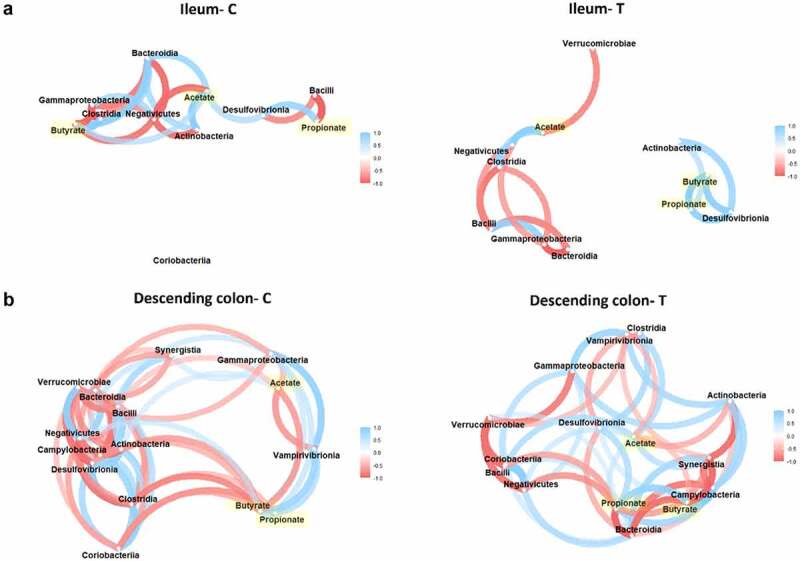
Associations between class taxa and SCFA concentrations in habitats and treatment-dependent fashion. Correlation networks illustrating the Spearman correlations between the SCFA produced under control (c) *vs*. ω-3 supplementation (t) and their corresponding class enrichment in opposed environments displayed in the luminal ileum (a) and descending colon (b). Gradient color, distance, and thickness of the lines were applied to nodes depending on coefficients of correlation. Only significant nodes that are upper to a coefficient of correlation of 0.5 or −0.5 are shown. Negative and positive correlations are denoted in shades of red and blue, respectively. SCFA nodes are highlighted in yellow.

Finally, to identify associations between bacterial abundance and SCFA concentration, a Spearman correlation matrix was generated, and significant associations were illustrated in a network format (Figure 7). The low microbiota diversity in the ileum (Figure 7a) showed limited associations between SCFA and taxa at the class level, though ω-3 PUFA supplementation (Ileum-T) modified the matrix, splitting it up into 2 distinct groups; acetate alone, on the one hand, and butyrate and propionate, which are positively associated (in blue) with the same taxa. More strikingly, in the descending colon, the significant increase of *Verrucomicrobiae* (*Akkermansia muciniphila*) and *Desulfovibrionia* (*Desulfovibrio*) observed under ω-3 PUFA supplementation (Descending colon-T), correlated positively with the observed rise of propionate (in blue, Figure 7b). The increase of *Negativicutes* members (*Veillonella, Dialister, Megasphaera*) was however correlated negatively (in red) with propionate production. The important decline of *Clostridia* members under ω-3 PUFA supplementation was not associated to the increase of propionate or decrease of butyrate, which instead correlated positively only with acetate (in blue). Therefore, Spearman correlations analysis displayed remarkable associations between metataxonomic traits and SCFA concentrations in a treatment and habitat-dependent fashion (Figure 7).

## Discussion

Ever increasing evidence indicates that ω-3 PUFAs, such as EPA and DHA, which are very rich in fish oil, confer health benefits that may be mediated, in part, by gut microbes through prebiotic effects.^9–14,21,22^ However, gut microbiota modulation associated with ω-3 PUFAs administration is still poorly understood in humans, primarily due to the lack of consensus in the choice of supplementation dose (~500 mg to 4 g per day), duration (~few days to 9 months), ratio of EPA/DHA and their formulation,^23^ study model (mice, human fecal samples from healthy or diseased phenotypes), and the inherent microbiota composition variability among subjects.^9–14,21,22^ Such limitations make the positioning of ω-3 PUFAs as a first-line prebiotic still unclear. To shed light on the mechanisms behind the interplay between the synergistic supplementation of the ω-3 PUFAs, EPA and DHA, within fish oil and gut microbiota functional changes, we conducted an explorative *in vitro* study. Moreover, to limit the risk of technical issues that can compromise the stability, reproducibility, and accuracy of the microbiota results, we designed a short-term study including 1-week control and 1-week supplementation with ω-3 PUFAs. Since complex gut niches (e.g., different intestinal/colonic regions and mucus-associated microbiota) are difficult to access *in vivo* in humans, we operated a sophisticated M-SHIME®, which gathers the complexity and dynamism of the human gastrointestinal tract and interface niches, as a closer proxy to human gastrointestinal physiology.^20^ This had the added benefit of allowing us to assess the direct effects of the ω-3 PUFAs independent of the role of host cells other than their capability of producing mucin.

The spatial positioning of gut microorganisms is important with respect to their functional role in the gut ecosystem.^24^ Regional differences across the longitudinal and axial direction are, however, not frequently studied. Our explorative study showed for the first time that a short-term ω-3 PUFA supplementation profoundly affects microbiota composition and main metabolic pathways in a complex gut habitat and niche-dependent fashion. Effects caused by the supplementation occurred predominantly in the transiting and floating mucus niche from the liquid part of the colon regions, defined as “floating luminal mucin”, which harbor mucolytic bacteria.^16,25,26^ The stratification between the outer layer of mucus and the luminal contents is important from the standpoint of host microbial mutualism.^27^ Although most bacterial species inhabit both compartments, there is a special niche in the mucus with differential nutrient resource utilization and strategies compared with the same species in the intestinal lumen.^25–27^ Indeed, a remarkable and significant bloom of the mucus specialist and metabolic health-promoting bacteria *Akkermansia muciniphila*^28^ was observed in all gut floating luminal mucin habitats following ω-3 PUFA supplementation. This observation was consistent with previous studies performed in human or mouse feces.^22,29,30^ Because the growth of *Akkermansia* is extremely pH dependent, the bacterium more abundantly colonized the distal regions of the colon where the pH is between 6.2 and 7 as previously observed *in vitro*.^31^ The stimulation of *Bifidobacterium* members often seen under ω-3 PUFAs,^22,29,30^ was instead, not observed in our case. In fact, for the first time, our study shows that most of the bacterial species with mucolytic properties,^27,32^ i.e. the *Actinobacteriota, Bifidobacterium longum*, and several *Firmicutes* including *Ruminococcus spp., Dorea longicatena, Oscillibacter spp*. and *Eubacterium hallii group* were significantly depleted in the different gut habitats of luminal floating mucin niche, contrary to the rise of the dominant *Akkermansia muciniphila*. Therefore, we wonder if the ω-3 PUFA supplementation promoted *Akkermansia* fitness with respect to the shift in relative abundance of other bacteria, or with respect to the mucin utilization. Future selection assays using limited concentration of mucin would help to conclude whether a competition exist for mucin glycan utilization and breakdown that benefits *Akkermansia muciniphila* at the expense of other mucolytic species under ω-3 PUFA supplementation.^25,26^ Moreover, since mucolytic bacteria can adapt their substrate preference from mucin glycans to dietary carbohydrates in relation to the types of nutrients available,^16,25,26^ it is also possible that the decrease of *Firmicutes* mucolytic bacteria was associated to the depletion of dietary carbohydrates, the main energy source of these gut microbes.

On the other hand, the outer mucus-associated microbiota (mucin-coated beads incorporated in the vessels) is described as a more robust microenvironment.^16^ It acts like a biofilm with only selective species able to attach selectively.^16^ Such physical attachment of the bacteria confers a competitive advantage in the face of the continuous flow through the gut. The robustness of outer mucus-attached bacteria was indeed confirmed in our work, with this microbiota niche remaining largely undisrupted following ω-3 PUFA supplementation. Conversely, the M-SHIME® is not set to integrate the inner mucus layer, which is firmly attached to the host intestinal epithelial cells and known to be virtually impenetrable to gut bacteria in a healthy state.^16,25,26^

On the other side of the interface, luminal non-mucus associated changes in microbiota composition were also observed. This unique study, by showing distinct changing signatures depending on different gut habitats and mucosal niches in response to ω-3 PUFAs, adds further complexity to the interpretation of the results in comparison with previous studies using different experimental models of the gut microbiota. In fact, the lack of consensus among studies precludes the drawing of definitive conclusions. For instance, in our work, the observed depletion of *Bacteroidota* in colon regions was in agreement with some studies performed in mice or on human fecal samples.^33–37^ This common trait was however contradicted by other reports.^11,12,22^ The overall decrease of *Actinobacteriota* in all gut habitats following ω-3 PUFAs corresponded well with the findings of Noriega *et al*^33^ but contradicted the one of Caesar *et al*.^29^ Still in the *Actinobacteriota* phylum, we also found that ω-3 PUFAs caused a strong and significant decrease of the genus *Collinsella* in all gut regions, in agreement with the clinical trial of Vijay *et al*.^11^ Interestingly, the same authors recently reported *Collinsella* to be increased in individuals with non-alcohol fatty liver disease (NAFLD).^38^ Given that NAFLD is known to be a risk factor for both insulin resistance and cardiometabolic disease, the authors suggested an important potential microbiome pathway by which ω-3 PUFAs produce a beneficial effect on health.^38^ With respect to the *Firmicutes* phylum, a significant decrease was observed in most of the gut habitats, and predominantly through the decrease of *Clostridia* members including *Agathobacter, Candidatus, Coprococcus, Eisenbergiella, Monoglobus, Flavonifractor, Hungatella, Intestinimonas, Lactonifactor* and *Negativibacillus*. Any generalized conclusion from these results remains difficult since some studies supported some of our observations,^22,39^ while others observed contradictory results, particularly with regard to the increase of *Coprococcus* and some members of the *Lachnospiraceae* family.^11,12^

Prebiotic properties consist of specific health-promoting changes of the microbiota composition that usually occur by altering microbial activities or abundances conferring benefits to the host physiology.^40,41^ Among the several metabolites released in the gut lumen by the microorganisms inhabiting it, SCFAs (fiber fermentation by-products) are of particular interest in the definition of prebiotic effects. In addition, EPA and DHA are known to positively correlate in *in vivo* studies with SCFA production, independently of fiber metabolism.^9,12^ Accordingly, we have shown in the present study a significant increase of propionate concentration in colon regions at the end of the 1-week ω-3 PUFAs administration. However, we did not observe an increase in butyrate, a trait that is often associated with ω-3 PUFA supplementation.^9,11,12^ This can be explained in our case by the observed relative luminal depletion of butyrate-producing bacterial taxa belonging to the *Lachnospiraceae* family of the *Firmicutes* phylum, such as *Coprococcus*.^11,42^ The evaluation of two gut microbiotas obtained in different seasons from the same individual in this study did not permit an extensive correlation-based analysis, but we did notice a remarkable association of *Akkermansia muciniphila* occurrence and members of the *Desulfovibrionia* class with increased propionate production. Such associations are consistent with the literature, acknowledging *Akkermansia muciniphila* as a propionate producer.^43^

The dynamics and fate of esterified EPA and DHA was studied here for the first time in an M-SHIME®. The physicochemical digestion of EPA/DHA-containing triacylglycerols starts in the stomach, but predominantly occurs in the duodenum with the release of pancreatic lipase, leading to free fatty acid and monoacylglycerol formation. Consequently, these triacylglycerol digestion products interact with bile salts to form micelles.^44,45^ This physiological and crucial step was successfully recreated in the M-SHIME®. Nonetheless, the subsequent step, leading to the absorption of free fatty acids and monoacylglycerols, is lacking in the M-SHIME® due to the lack of intestinal epithelial cells, and this represents the main limitation of this explorative model.^23^ Therefore, the bioavailability/host-dependent absorption of the supplement could not be mimicked. For this reason, and compared to other studies,^9–14,21–23^ we added in the stomach vessels the minimum recommended dose (by the manufacturer) of 990 mg/day each, EPA, and DHA (Quell Fish Oil®, Douglas Laboratories, Ontario, Canada). We observed a gradual but low accumulation of free EPA and DHA over time due to the longitudinal and semi-continuous transfer of the ω-3 PUFA supplement and digestive secretion across the different gut sections from stomach to descending colon.

Finally, on top of the regional and niche variations, which we recreated in the M-SHIME®, a substantial seasonal variation of the gut microbiota is often seen and underestimated in most clinical trials.^46,47^ Such “seasonal” variation also implies the possible change of diet over time, health status, or any other environmental exposures that can affect the gut microbiota as well. For this reason, we evaluated here whether the collection from the same donor of the fecal microbiota in two different (and opposite) seasons (summer and winter) affected the response to the ω-3 PUFAs. We observed that the distinct microbiota composition between summer and winter, however, developed similar microbial community profile changes in response to ω-3 PUFAs. Thus, it appears that the overall direct effects of ω-3 PUFA supplementation on the gut microbiome may be independent of its seasonal variation, though the assessment of more individuals will be required to confirm this.

As a future perspective, further investigations in the same M-SHIME® set up, performed by increasing the number of microbiotas to inoculate, will be required for the assessment of whether inter-individual variabilities would reinforce the understanding of regionalized and niche-dependent effects of fish oil-like ω-3 PUFA supplementation. Extensive correlative analyses, combining, for example, targeted lipidomics profiling of EPA and DHA-derived metabolites with microbiota composition data, would also increase the impact of the use of the M-SHIME® in studies such as the present one (manuscript in preparation). Such an approach would produce further evidence that ω-3 PUFAs can be metabolized by particular members of the gut microbiota, resulting therefore in the production of potential bioactive metabolites, possibly similar to those produced by host cells.^48^ Finally, in the ideal perspective of coming closer to human gastrointestinal physiology, the potency of the M-SHIME® should be coupled with the host counterpart.^49^ Combining SHIME® digestive effluents with cell cultures of the intestinal epithelium and goblet cells (inner mucus layer) or organoids expressing all cell types would provide mechanistic information on the propensity of epithelial cells to change the function and composition of the microbiota when treated with ω-3 PUFAs.^50^

In conclusion, we have provided here unprecedented evidence that, in an M-SHIME® setting reproducing both the lumen associated with floating mucin and the outer mucus niches of the human gut, a fish oil-like supplement rich in EPA and DHA-containing triacylglycerols is capable of producing strong metataxonomic, and corresponding functional, changes of the gut microbiota, in a habitat and niche, but not a seasonal, dependent manner. Such changes, strongly suggestive of potential host cell-independent and gut microbiota-mediated beneficial effects of ω-3 PUFAs, may explain at least part of the wealth of benefits on metabolism and inflammation suggested by preclinical and clinical studies with this type of supplements, for which our data support a possible new role as prebiotics.

## Methods

### Fermentation system

The TWIN-M-SHIME® (Prodigest, Belgium) is a simulator of the human intestinal microbial ecosystem with two anaerobic SHIME® units operated dynamically in parallel, in semi-continuous mode.^20^ In this study, a SHIME® unit consisted of a stomach/proximal small intestine vessel to reproduce gastric digestion of a standardized nutritional medium pancreatic/bile juice delivery, followed by an ileum, ascending, transverse and descending colon vessels. Two microbiotas from the same fecal donor (healthy men, 47 y old, without antibiotic history) were tested in duplicate at different seasons (summer and winter, n = 4). For experimental reproducibility, fecal duplicate, one in each SHIME® unit, inoculated the distinct colonic regions to capture region-dependent microbiota composition and metabolism. The procedure for fecal inoculum preparation was previously described for the colonic regions.^51^ To establish an ileal microbial community at low biomass concentration, more representative of the human physiology, a 100-μL microbial inoculum was taken from the ascending colon and diluted 50 times with an anaerobic buffer prior to inoculate the ileum vessel. The nutritional SHIME® medium classically used was supplemented in this set-up, with simple sugars 0.5 g L^−1^ each, i.e., glucose, fructose, galactose, maltose, and sucrose to enhance the growth of bacteria usually found in the ileum. Floating mucin was also present in the luminal habitats. Outer mucus-associated microbiotas of the ileum, ascending, transverse and descending colons were mimicked through the incorporation of microcosms (AnoxKaldnes K1 carrier, Lund, Sweden) coated with type II porcine mucin-agar containing MUC2 gel-forming mucin (Sigma-aldrich, St. Louis, US). General functioning of the system, mucin carrier replacement and media composition have been presented in Roussel *et al*.^51^ The overall TWIN-M-SHIME® fermentation was performed for 28 d including a 14-d stabilization period, a 7-d control diet followed by a 7-d ω-3 fish oil treatment. Treatment consisted in the supplementation of 990 mg EPA and 990 mg DHA esterified in triacylglycerols (Quell Fish Oil®, Douglas Laboratories, Ontario, Canada)/day/replicate in the SHIME® stomach. Both periods, control and treatment were supplemented with an emulsifier containing 0.38% (w/v) lecithin (Sigma-Aldrich, St. Louis, US) to help solubilizing the oil. All vessels were protected from light source to prevent photo-oxidation of the ω-3 PUFAs. SHIME® suspensions from ileum and colon vessels were regularly sampled, centrifuged at 4°C, 18 000 *× g* for 8 minutes for subsequent SCFA and DNA analysis, and stored at −20°C (Supplementary data Figure S5). Bioreactors were carefully opened under N_2_ flush every 3 d to collect half of the mucin microcosms to reproduce the physiological desquamation process of the mucosal layer.^52^ The removed beads were used for sampling and replaced by fresh beads. Aliquots of 125 mg mucus from the ileum vessel, and 250 mg from the colon vessels were taken with a mini sampler spoon (Bel Art^TM^ Scienceware^TM^, Thermo Fisher Scientific, Waltham, US) and stored at −20°C prior to DNA extraction. Samples for lipids EPA and DHA analysis were taken regularly for kinetics, centrifuged at 4°C, 18 000 *× g* for 8 minutes, and stored at −80°C (Supplementary data Figure S5).

### DNA extraction

SHIME® suspensions from ileum and colon vessels (500 μL) were stained with 1.25 μL of propidium monoazide (PMA, 50 μM) (Biotium, Fremont, Canada) to inactivate dead bacterial DNA, as previously described.^53^ DNA was pelleted (8 minutes, 18 000 *× g*, 4°C), and extracted according to Geirnaert *et al*.^54^ In the mucus microcosm samples, an additional step was performed to increase DNA yield and break the disulfide bonds from the mucins. Sputolysin 0.1 M (250 μL, Sigma-Aldrich, St. Louis, US) was added to 125 mg mucus sample from ileum or 250 mg mucus from colons. Samples were incubated in a water bath for 30 minutes at 37°C prior to the DNA extraction procedure. DNA extracts were eluted in 1X TE buffer (Tris and EDTA) and stored at −20°C until sequencing. The quality of DNA was analyzed by gel electrophoresis (1.2% w/v agarose) (Life technologies, Madrid, Spain). Concentrations were measured by the Qubit (Thermo Fisher Scientific, Waltham, US) and the DNA were stored at −20°C, until 16S rDNA library preparation.

### Library preparation

The QIAseq 16S Region Panel protocol in conjunction with the QIAseq 16S/ITS 384-Index I (Sets A, B, C, D) kit (Qiagen, Hilden, Germany) were used for amplification and indexing of the V3-V4 region of the 16S rRNA gene (341 F-805 R) for all DNA samples. The 16S metagenomic libraries were qualified by Agilent High Sensitivity DNA Kit (Agilent, Palo Alto, US) using a Bioanalyser to verify the amplicon size (expected size ~660 bp) and quantified with both a Quant-iT PicoGreen dsDNA Kit (Thermo Fisher Scientific, Waltham, US), and a Qubit (Thermo Fisher Scientific, Waltham, US). Libraries were then normalized and pooled to 4 nM, denatured and diluted to a final concentration of 10 pM.

### Microbial community analysis

Sequencing was performed using the MiSeq 600 cycles Reagent Kit V3 (Illumina, San Diego, US) by an Illumina MiSeq System (Illumina, San Diego, US). All 16S sequencing data has been deposited in the NCBI with SRA accession number PRJNA836582.

*Bioinformatics analysis*. The Divisive Amplicon Denoising Algorithm (DADA2) workflow implemented in the dada2 R package 1.18.0 was employed to identify Amplicons Sequences Variants (ASVs).^55^ The reads were merged if they overlapped precisely, and an ASVs table was constructed, recording the number of times each ASV was observed in each sample. Default parameters were used to estimate error rates using *learnErrors*, and chimeras were removed using *removeBimeraDenova* (method = ”consensus”).^55^ ASV sequences were assigned taxonomy using the most recent SILVA taxonomic database (SILVA SSURef 138.1 NR, March 2021) as a reference dataset.^56^ A phyloseq data object was created using the *phyloseq* package in R.^57^ Unassigned taxa and singletons were removed. Sequences detected in less than 5% of all samples were filtered out.^58^ Rarefaction curves were constructed to ensure that the samples were sequenced at sufficient depth.^58,59^ To deal with differences in sampling depth, the data were rescaled to proportions for further analysis.^60^ Lack of species assignation with SILVA database, a manual refinement was performed by querying of sequences against another official representative meta database: the RDP SeqMatch classifier tool, Michigan State University. Query sequences were assigned taxonomy at the species level based on database hits if identify matched at sup. 99%. In case of inconsistencies between the RDP SeqMatch tool and NCBI BLAST, no species level classification was mentioned.

*Statistical analysis of amplicon data*. All statistical analyses were performed in R 4.0.4 (R Core Team, 2021). All formal hypothesis tests were conducted on the 5% significance level. The evolution of the microbial community α-diversity between conditions was followed by computing the Shannon index using vegan package 2.5–7^58,61^ and ggpubr 0.4.0^62^ to compare means statistics. To highlight differences in microbial community composition between conditions, ordination, and clustering techniques were applied and visualized with ggplot2 3.3.5. The influence of the treatments, gut habitats, donors, and periods was determined by applying a db-RDA using the abundance-based jaccard distance as a response variable (vegan 2.5–7).^58,61^ The factor treatment (control, ω-3 PUFA supplementation) was used as a constraint with the effect of gut regions, replicates, and time being partially out. Interpretation of the results was preceded by a permutation test of the RDA results to confirm that a linear relationship exists between the response data and the exploratory variables. The constrained fraction of the variance explained by the exploratory variables was adjusted by applying Ezekiel’s formula.^62^ This procedure was repeated on species and genus levels. On the genus level, weighed averages of genera abundances were a *posteriori* added to the ordination plot using the wascores function in vegan.^58^ To confirm the trends, observed data were clustered by means of an Unweighted Pair-Grouped Method using arithmetic Averages (UPGMA) clustering method (cluster 2.1.0).^59^ The significance of the observed group separation between gut region, donor, and period in the PCoA was assessed with a Permutational Multivariate Analysis of Variance (PERMANOVA) using distance matrixes (vegan 2.5–7).^58,61^ Prior to this formal hypothesis testing, the assumption of similar multivariate dispersions was evaluated. To find statistically significant differences in species/genera abundance between control and ω-3 PUFA supplementation, the DESeq2 package 1.30.1 was applied.^57,59^ The factors treatment, period, gut region, and donor were used in the design of LRT formula. Statistical differences between the control and ω-3 PUFA supplementation across gut habitats were determined using a Wald Test. P-values were adjusted for multiple testing using the Benjamini-Hochberg procedure. Significant differences were visualized in a volcanoplot, showing the -log10(adjusted p-value) as a function of the shrunken log2 Fold Change.^59,63^ In addition, the log transformed normalized pseudocounts of the most abundant genera and species displaying the most pronounced shrunken log2 Fold Changes, retrieved by the plotCounts function, were displayed in box plots, with a fill color depending on the gut region and time.^59,63^ Spearman’s correlation matrix and networks were constructed with the corrr package 0.4.3.

### SCFA production

SHIME® effluents from ileum and colon vessels (125 μL) were centrifuged at 18 000 *× g* for 8 minutes at 4°C. SCFA were extracted with diethyl ether and analyzed through a gas chromatograph equipment coupled to a flame ionization detector.^64^ SCFA concentration was expressed in mM. Statistical analysis was performed by using the Kruskal–Wallis rank sum test, followed by Pairwise Wilcoxon Rank Sum Test with Holm correction.

### Lipid Extraction and HPLC-MS/MS for the analysis of EPA/DHA

Lipids were extracted from the SHIME® suspensions (stomach, proximal intestine, ileum, and colons) as previously described.^65–67^ Each sample (40 μL) were finally injected onto an HPLC column (Kinetex C8, 150 × 2.1 mm, 2.6 μm, Phenomenex) and eluted at a flow rate of 400 μL/minutes using a discontinuous gradient of solvent A and solvent B .^65–67^ Quantification of EPA/DHA was carried out by HPLC interfaced with the electrospray source of a Shimadzu 8050 triple quadrupole mass spectrometer and using multiple reaction monitoring in positive ion mode for the compounds and their deuterated homologs.^65–67^

## Supplementary Material

Supplemental Material

## Data Availability

Raw 16S rRNA gene sequence data were made publicly available online through the Sequence Read Archive (SRA) portal of NCBI under accession number PRJNA836582.

## References

[1] Zárate R, El Jaber-Vazdekis N, Tejera N, Pérez JA, Covadonga R. Significance of long chain polyunsaturated fatty acids in human health. Clin Trans Med. 2017;6(1). doi:10.1186/s40169-017-0153-6. PMC5532176 28752333

[2] Saini RK, Prasad P, Sreedhar RV, Akhilender Naidu K, Shang X, Keum Y-S. Omega−3 polyunsaturated fatty acids (pufas): emerging plant and microbial sources, oxidative stability, bioavailability, and health benefits—a review. Antioxidants. 2021;10(10):1627. doi:10.3390/antiox10101627. 34679761 PMC8533147

[3] Liao J, Xiong Q, Yin Y, Ling Z, Chen S. The effects of fish oil on cardiovascular diseases: systematical evaluation and recent advance. Front Cardiovasc Med. 2022;8:802306. 10.3389/fcvm.2021.802306 35071366 PMC8767101

[4] Dyall SC. Long-chain omega-3 fatty acids and the brain: a review of the independent and shared effects of EPA, DPA and DHA. Front Aging Neurosci. 2015;7:52. 10.3389/fnagi.2015.00052. 25954194 PMC4404917

[5] Borsini A, Nicolaou A, Camacho-Muñoz D, Kendall AC, Di Benedetto MG, Giacobbe J, Su K-P, Pariante CM. Omega-3 polyunsaturated fatty acids protect against inflammation through production of LOX and CYP450 lipid mediators: relevance for major depression and for human hippocampal neurogenesis. Mol Psychiatry. 2021;26(11):6773–20. 10.1038/s41380-021-01160-8 34131267 PMC8760043

[6] Gao H, Geng T, Huang T, Zhao Q. Fish oil supplementation and insulin sensitivity: a systematic review and meta-analysis. Lipids Health Dis. 2017;16(1):131. 10.1186/s12944-017-0528-0. 28673352 PMC5496233

[7] Natto ZS, Yaghmoor W, Alshaeri HK, Van Dyke TE. Omega-3 fatty acids effects on inflammatory biomarkers and lipid profiles among diabetic and cardiovascular disease patients: a systematic review and meta-analysis. Sci Rep. 2019;9(1):18867. 10.1038/s41598-019-54535-x. 31827125 PMC6906408

[8] Freitas RDS, Campos MM. Protective effects of omega-3 fatty acids in cancer-related complications. Nutrients. 2019;11(5):945. 10.3390/nu11050945. PMC6566772 31035457

[9] Costantini L, Molinari R, Farinon B, M®erendino N. Impact of omega-3 fatty acids on the gut microbiota. Int J Mol Sci. 2017; 18(12): 2645. 10.3390/ijms18122645 PMC5751248 29215589

[10] Fu Y, Wang Y, Gao H, Li D, Jiang R, Ge L, Tong C, Xu K. Associations among dietary omega-3 polyunsaturated fatty acids, the gut microbiota, and Intestinal Immunity. Mediators Inflamm. 2021;2021:8879227. 10.1155/2021/8879227. 33488295 PMC7801035

[11] Vijay A, Astbury S, Le Roy C, Spector TD, Valdes AM. The prebiotic effects of omega-3 fatty acid supplementation: a six-week randomised intervention trial. Gut Microbes. 2021;13(1):1–11. 10.1080/19490976.2020.1863133. PMC7781624 33382352

[12] Menni C, Zierer J, Pallister T, Jackson, M.A., Long, T., Mohney, R.P., Steves, C.J., Spector, T.D., Valdes, A.M. Omega-3 fatty acids correlate with gut microbiome diversity and production of N-carbamylglutamate in middle aged and elderly women. Sci Rep. 2017;7(1):11079. 10.1038/s41598-017-10382-2 28894110 PMC5593975

[13] Zheng D, Liwinski T, Elinav E. Interaction between microbiota and immunity in health and disease. Cell Res. 2020;30(6):492–506. 10.1038/s41422-020-0332-7. 32433595 PMC7264227

[14] Nana G, Mitra S, Watson H, Young C, Wood HM, Perry SL, Race AD, Quirke P, Toogood GJ, Loadman PM, et al. Luminal bioavailability of orally administered ω-3 PUFAs in the distal small intestine, and associated changes to the ileal microbiome, in humans with a temporary ileostomy. J Nutr. 2021;151(8):2142–2152. 10.1093/jn/nxab113. 34036331 PMC8349127

[15] Ringel Y, Maharshak N, Ringel-Kulka T, Wolber EA, Sartor RB, Carroll IM. High throughput sequencing reveals distinct microbial populations within the mucosal and luminal niches in healthy individuals. Gut Microbes. 2015;6(3):173–181. 10.1080/19490976.2015.1044711. 25915459 PMC4615648

[16] Paone P, Cani PD. Mucus barrier, mucins and gut microbiota: the expected slimy partners? Gut. 2020;69(12):2232–2243. 10.1136/gutjnl-2020-322260. 32917747 PMC7677487

[17] Johansson ME, Hansson GC. Immunological aspects of intestinal mucus and mucins. Nat Rev Immunol. 2016;16(10):639–649. 10.1038/nri.2016.88. 27498766 PMC6435297

[18] Escoula Q, Bellenger S, Narce M, Bellenger J. Docosahexaenoic and eicosapentaenoic acids prevent altered-Muc2 secretion induced by palmitic acid by alleviating endoplasmic reticulum stress in LS174T goblet cells. Nutrients. 2019;11(9):2179. 10.3390/nu11092179. PMC6770956 31514316

[19] Rifkin SB, Sen A, Turgeon DK, Chan R, Ruffin MT, Brenner DE, Schloss PD, Djuric Z. Increased *Akkermansia* abundance is associated with increased colonic mucosal ω-3 fatty acids and decreased colonic mucosal PGE_2_ concentrations following healthy dietary pattern interventions. MedRxiv. 2021. doi:10.1101/2021.10.11.21264748.

[20] Van de Wiele T, Van den Abbeele P, Ossieur W, Possemiers S, Marzorati M. The Simulator of the Human Intestinal Microbial Ecosystem (SHIME®). Verhoeckx K, Cotter P, López-Expósito I, Kleiveland C, Lea T, Mackie A, et al.editors. The impact of food bioactives on health: in vitro and ex vivo models. Cham (CH). Springer International Publishing; 2015. p. 305–317.

[21] Rajkumar H, Mahmood N, Kumar M, Varikuti SR, Challa HR, Myakala SP. Effect of probiotic (VSL#3) and omega-3 on lipid profile, insulin sensitivity, inflammatory markers, and gut colonization in overweight adults: a randomized, controlled trial. Mediators Inflamm. 2014;2014:348959. doi:10.1155/2014/348959. 24795503 PMC3984795

[22] Watson H, Mitra S, Croden FC, Taylor M, Wood HM, Perry SL, Spencer JA, Quirke P, Toogood GJ, Lawton CL, et al. A randomised trial of the effect of omega-3 polyunsaturated fatty acid supplements on the human intestinal microbiota. Gut. 2018;67(11):1974–1983. doi:10.1136/gutjnl-2017-314968. 28951525

[23] Maki KC, Dicklin MR. Strategies to improve bioavailability of omega-3 fatty acids from ethyl ester concentrates. Curr Opin Clin Nutr Metab Care. 2019;22(2):116–123. doi:10.1097/MCO.0000000000000537. 30550388

[24] Thursby E, Juge N. Introduction to the human gut microbiota. Biochem J. 2017;474(11):1823–1836. doi:10.1042/BCJ20160510. 28512250 PMC5433529

[25] Berkhout MD, Plugge CM, Belzer C. How microbial glycosyl hydrolase activity in the gut mucosa initiates microbial cross-feeding. Glycobiology. 2022;32(3):182–200. 10.1093/glycob/cwab105. 34939101 PMC8966484

[26] Belzer C. Nutritional strategies for mucosal health: the interplay between microbes and mucin glycans. Trends Microbiol. 2022;30(1):13–21. 10.1016/j.tim.2021.06.003. 34217596

[27] Li H, Limenitakis JP, Fuhrer T, Geuking MB, Lawson MA, Wyss M, Brugiroux S, Keller I, Macpherson JA, Rupp S, et al. The outer mucus layer hosts a distinct intestinal microbial niche. Nat Commun. 2015;6(1):8292. doi:10.1038/ncomms9292. 26392213 PMC4595636

[28] Depommier C, Everard A, Druart C, Maiter D, Thissen J-P, Loumaye A, Hermans MP, Delzenne NM, de Vos WM, Cani PD, et al. Serum metabolite profiling yields insights into health promoting effect of A. muciniphila in human volunteers with a metabolic syndrome. Gut Microbes. 2021;13(1):1994270. doi:10.1080/19490976.2021.1994270. 34812127 PMC8632301

[29] Caesar R, Tremaroli V, Kovatcheva-Datchary P, Cani PD, Bäckhed F. Crosstalk between gut microbiota and dietary lipids aggravates WAT Inflammation through TLR signaling. Cell Metab. 2015;22(4):658–668. doi:10.1016/j.cmet.2015.07.026. 26321659 PMC4598654

[30] Kaliannan K, Wang B, Li XY, Kim KJ, Kang JX. A host-microbiome interaction mediates the opposing effects of omega-6 and omega-3 fatty acids on metabolic endotoxemia. Sci Rep. 2015;5(1):11276. doi:10.1038/srep11276. 26062993 PMC4650612

[31] Van Herreweghen F, den Abbeele P V, De Mulder T, De Weirdt R, Geirnaert A, Hernandez-Sanabria E, Vilchez-Vargas R, Jauregui R, Pieper DH, Belzer C, et al. In vitro colonisation of the distal colon by Akkermansia muciniphila is largely mucin and pH dependent. Benef Microbes. 2017;8(1):81–96. 10.3920/BM2016.0013. 27824274

[32] Png CW, Lindén SK, Gilshenan KS, Zoetendal EG, McSweeney CS, Sly LI, McGuckin MA, Florin THJ. Mucolytic bacteria with increased prevalence in IBD mucosa augment in vitro utilization of mucin by other bacteria. Am J Gastroenterol. 2010;105(11):2420–2428. doi:10.1038/ajg.2010.281. 20648002

[33] Noriega BS, Sanchez-Gonzalez MA, Salyakina D, Coffman J. Understanding the impact of Omega-3 rich diet on the gut microbiota. Case Rep Med. 2016;2016:3089303. doi:10.1155/2016/3089303. 27065349 PMC4808672

[34] Hildebrandt MA, Hoffmann C, Sherrill-Mix SA, Keilbaugh SA, Hamady M, Chen Y, Knight R, Ahima RS, Bushman F, Wu GD, et al. High-fat diet determines the composition of the murine gut microbiome independently of obesity. Gastroenterology. 2009;137(5):1716–24.e242. doi:10.1053/j.gastro.2009.08.042. 19706296 PMC2770164

[35] Devkota S, Wang Y, Musch MW, Leone V, Fehlner-Peach H, Nadimpalli A, Antonopoulos DA, Jabri B, Chang EB. Dietary-fat-induced taurocholic acid promotes pathobiont expansion and colitis in Il10-/- mice. Nature. 2012;487(7405):104–108. doi:10.1038/nature11225. 22722865 PMC3393783

[36] Liu T, Hougen H, Vollmer AC, Hiebert SM. Gut bacteria profiles of mus musculus at the phylum and family levels are influenced by saturation of dietary fatty acids. Anaerobe. 2012;18(3):331–337. doi:10.1016/j.anaerobe.2012.02.004. 22387300

[37] Mokkala K, Röytiö H, Munukka E, Pietilä, S., Ekblad, U., Rönnemaa, T., Eerola, E., Laiho, A., Laitinen, K. Gut microbiota richness and composition and dietary intake of overweight pregnant women are related to serum zonulin concentration, a marker for intestinal permeability. J Nutr. 2016;146(9):1694–1700. doi:10.3945/jn.116.235358. 27466607

[38] Astbury S, Atallah E, Vijay A, Aithal GP, Grove JI, Valdes AM. Lower gut microbiome diversity and higher abundance of proinflammatory genus *Collinsella* are associated with biopsy-proven nonalcoholic steatohepatitis. Gut Microbes. 2020;11(3):569–580. doi:10.1080/19490976.2019.1681861. 31696774 PMC7524262

[39] Yu HN, Zhu J, Pan WS, Shen SR, Shan WG, Das UN. Effects of fish oil with a high content of n-3 polyunsaturated fatty acids on mouse gut microbiota. Arch Med Res. 2014;45(3):195–202. doi:10.1016/j.arcmed.2014.03.008. 24681186

[40] Gibson GR, Hutkins R, Sanders ME, Prescott, S.L., Reimer, R.A., Salminen, S.J., Scott, K., Stanton, C., Swanson, K.S., Cani, P.D., Verbeke, K. Expert consensus document: the international scientific association for probiotics and prebiotics (ISAPP) consensus statement on the definition and scope of prebiotics. Nat Rev Gastroenterol Hepatol. 2017;14(8):491–502. doi:10.1038/nrgastro.2017.75. 28611480

[41] Neri-Numa IA, Pastore GM. Novel insights into prebiotic properties on human health: a review. Food Res Int. 2020;131:108973. doi:10.1016/j.foodres.2019.108973. 32247494

[42] Louis P, Flint HJ. Formation of propionate and butyrate by the human colonic microbiota. Environ Microbiol. 2017;19(1):29–41. doi:10.1111/1462-2920.13589. 27928878

[43] El Hage R, Hernandez-Sanabria E, Calatayud Arroyo M, Props R, Van de Wiele T. Propionate-producing consortium restores antibiotic-induced dysbiosis in a dynamic *in vitro* model of the human intestinal microbial ecosystem. Front Microbiol. 2019;10(10):1206. doi:10.3389/fmicb.2019.01206. 31214145 PMC6554338

[44] Wilde PJ, Chu BS. Interfacial & colloidal aspects of lipid digestion. Adv Colloid Interface Sci. 2011;165(1):14–22. doi:10.1016/j.cis.2011.02.004. 21377138

[45] McClements DJ, Decker EA, Park Y. Controlling lipid bioavailability through physicochemical and structural approaches. Crit Rev Food Sci Nutr. 2008;49(1):48–67. doi:10.1080/10408390701764245. 18949598

[46] Vandeputte D, De Commer L, Tito RY, Kathagen, G., Sabino, J., Vermeire, S., Faust, K. and Raes, J. Temporal variability in quantitative human gut microbiome profiles and implications for clinical research. Nat Commun. 2021;12(1):6740. doi:10.1038/s41467-021-27098-7. 34795283 PMC8602282

[47] Koliada A, Moseiko V, Romanenko M, Piven L, Lushchak O, Kryzhanovska N, Guryanov V, Vaiserman A. Seasonal variation in gut microbiota composition: cross-sectional evidence from Ukrainian population. BMC Microbiol. 2020;20(1):100. doi:10.1186/s12866-020-01786-8. 32316935 PMC7175530

[48] Shama S, Liu W. Omega-3 fatty acids and gut microbiota: a reciprocal interaction in nonalcoholic fatty liver disease. Dig Dis Sci. 2020;65(3):906–910. 10.1007/s10620-020-06117-5. 32036510 PMC7145364

[49] Beterams A, De Paepe K, Maes L, Wise IJ, De Keersmaecker H, Rajkovic A, Laukens D, Van de Wiele T, Calatayud Arroyo M. Versatile human in vitro triple coculture model coincubated with adhered gut microbes reproducibly mimics pro-inflammatory host-microbe interactions in the colon. FASEB J. 2021;35(12):e21992. doi:10.1096/fj.202101135R. 34719821

[50] Durkin LA, Childs CE, Calder PC. Omega-3 polyunsaturated fatty acids and the intestinal epithelium-a review. Foods. 2021;10(1):199. doi:10.3390/foods10010199. 33478161 PMC7835870

[51] Roussel C, De Paepe K, Galia W, De Bodt J, Chalancon S, Leriche F, Ballet N, Denis S, Alric M, Van de Wiele T, et al. Spatial and temporal modulation of enterotoxigenic E. coli H10407 pathogenesis and interplay with microbiota in human gut models. BMC Biol. 2020;18(1):141. doi:10.1186/s12915-020-00860-x. 33054775 PMC7559199

[52] den Abbeele P V, Roos S, Eeckhaut V, MacKenzie DA, Derde M, Verstraete W, Marzorati M, Possemiers S, Vanhoecke B, Van Immerseel F, et al. Incorporating a mucosal environment in a dynamic gut model results in a more representative colonization by lactobacilli. Microb Biotechnol. 2012;5(1):106–115. doi:10.1111/j.1751-7915.2011.00308.x. 21989255 PMC3815277

[53] Roussel C, Galia W, Leriche F, Chalancon S, Denis S, Van de Wiele T, Blanquet-Diot S. Comparison of conventional plating, PMA-qPCR, and flow cytometry for the determination of viable enterotoxigenic *Escherichia coli* along a gastrointestinal in vitro model. Appl Microbiol Biotechnol. 2018;102(22):9793–9802. doi:10.1007/s00253-018-9380-z. 30238141

[54] Geirnaert A, Wang J, Tinck M, Steyaert A, Van den Abbeele P, Eeckhaut V, Vilchez-Vargas R, Falony G, Laukens D, De Vos M, et al. Interindividual differences in response to treatment with butyrate-producing butyricicoccus pullicaecorum 25-3T studied in an in vitro gut model. FEMS Microbiol Ecol. 2015;91. doi:10.1093/femsec/fiv054. 25999470

[55] Callahan BJ, Mcmurdie PJ, Rosen MJ, Han AW, Johnson AA, Holmes SP. DADA2: high-resolution sample inference from Illumina amplicon data. Na Methods. 2016;13(7):581–583. doi:10.1038/nmeth.3869. PMC4927377 27214047

[56] McLaren MR, Callahan BJ. Silva 138.1 prokaryotic SSU taxonomic training data formatted for DADA2 [Data set]. Zenodo. 2021: doi:10.5281/zenodo.4587955.

[57] McMurdie PJ, Holmes S. Waste not, want not: why rarefying microbiome data is inadmissible. PLoS Comput Biol. 2014;10(4):e1003531. doi:10.1371/journal.pcbi.1003531. 24699258 PMC3974642

[58] Oksanen J, Blanchet G, Friendly M, Kindt R, Legendre P, McGlinn D, Minchin PR, O’Hara RB, Simpson GL, Solymos P, et al. Vegan: community ecology package. R package version 2.4–0. Tech. rep; 2022. Available from https://cran.r-project.org/web/packages/vegan/vegan.pdf. Accessed 28 July 2022.

[59] Love MI, Huber W, Anders S. Moderated estimation of fold change and dispersion for RNA-seq data with DESeq2. Genome Biol. 2014;15(12):550. doi:10.1186/s13059-014-0550-8. 25516281 PMC4302049

[60] Schloss PD, Westcott SL. Assessing and improving methods used in operational taxonomic unit-based approaches for 16S rRNA gene sequence analysis. Appl Environ Microbiol. 2011;77(10):3219–3226. doi:10.1128/AEM.02810-10. 21421784 PMC3126452

[61] Ramette A. Multivariate analyses in microbial ecology. FEMS Microbiol Ecol. 2007;62(2):142–160. doi:10.1111/j.1574-6941.2007.00375.x. 17892477 PMC2121141

[62] Wickham H, Averick M, Bryan J, Chang W, McGowan LD, François R, Grolemund G, Hayes A, Henry L, Hester J, et al. Welcome to the tidyverse. J Open Source Softw. 2019;4(43):1686. 10.21105/joss.01686.

[63] De Paepe K, Verspreet J, Verbeke K, Raes J, Courtin CM, Van de Wiele T. Introducing insoluble wheat bran as a gut microbiota niche in an in vitro dynamic gut model stimulates propionate and butyrate production and induces colon region specific shifts in the luminal and mucosal microbial community. Environ Microbiol. 2018;20(9):3406–3426. doi:10.1111/1462-2920.14381. 30126070

[64] Roussel C, Chabaud S, Lessard-Lord J, Cattero V, Pellerin FA, Feutry P, Bochard V, Bolduc S, Desjardins Y. UPEC colonic-virulence and urovirulence are blunted by proanthocyanidins-rich cranberry extract microbial metabolites in a gut model and a 3D tissue-engineered urothelium. Microbiol. Spect 2022;e0243221. doi:10.1128/spectrum.02432-21. PMC9603664 35972287

[65] Manca C, Boubertakh B, Leblanc N, Deschênes T, Lacroix S, Martin C, Houde A, Veilleux A, Flamand N, Muccioli GG, et al. Germ-free mice exhibit profound gut microbiota-dependent alterations of intestinal endocannabinoidome signaling. J Lipid Res. 2020;61(1):70–85. doi:10.1194/jlr.RA119000424. 31690638 PMC6939599

[66] Lacroix S, Pechereau F, Leblanc N, Boubertakh B, Houde A, Martin C, Flamand N, Silvestri C, Raymond F, Di Marzo V, et al. Rapid and concomitant gut microbiota and endocannabinoidome response to diet-induced obesity in mice. msystems. 2019;4(6):e00407–e00419. doi:10.1128/msystems.00407-19. 31848310 PMC6918026

[67] Depommier C, Vitale RM, Iannotti FA, Silvestri C, Flamand N, Druart C, Everard A, Pelicaen R, Maiter D, Thissen JP, et al. Beneficial effects of akkermansia muciniphila are not associated with major changes in the circulating endocannabinoidome but linked to higher mono-palmitoyl-glycerol levels as new pparα agonists. Cells. 2021;10(1):185. doi:10.3390/cells10010185. 33477821 PMC7832901

