# Supplementary material for: Short-term supplementation with ω-3 polyunsaturated fatty acids modulates primarily mucolytic species from the gut luminal mucin niche in a human fermentation system
Source: Gut Microbes. 2022 Sep 15;14(1):2120344. doi: 10.1080/19490976.2022.2120344 (PMC9481098; doi:10.1080/19490976.2022.2120344)
Supplement: Supplemental Material [file KGMI_A_2120344_SM3680.docx]

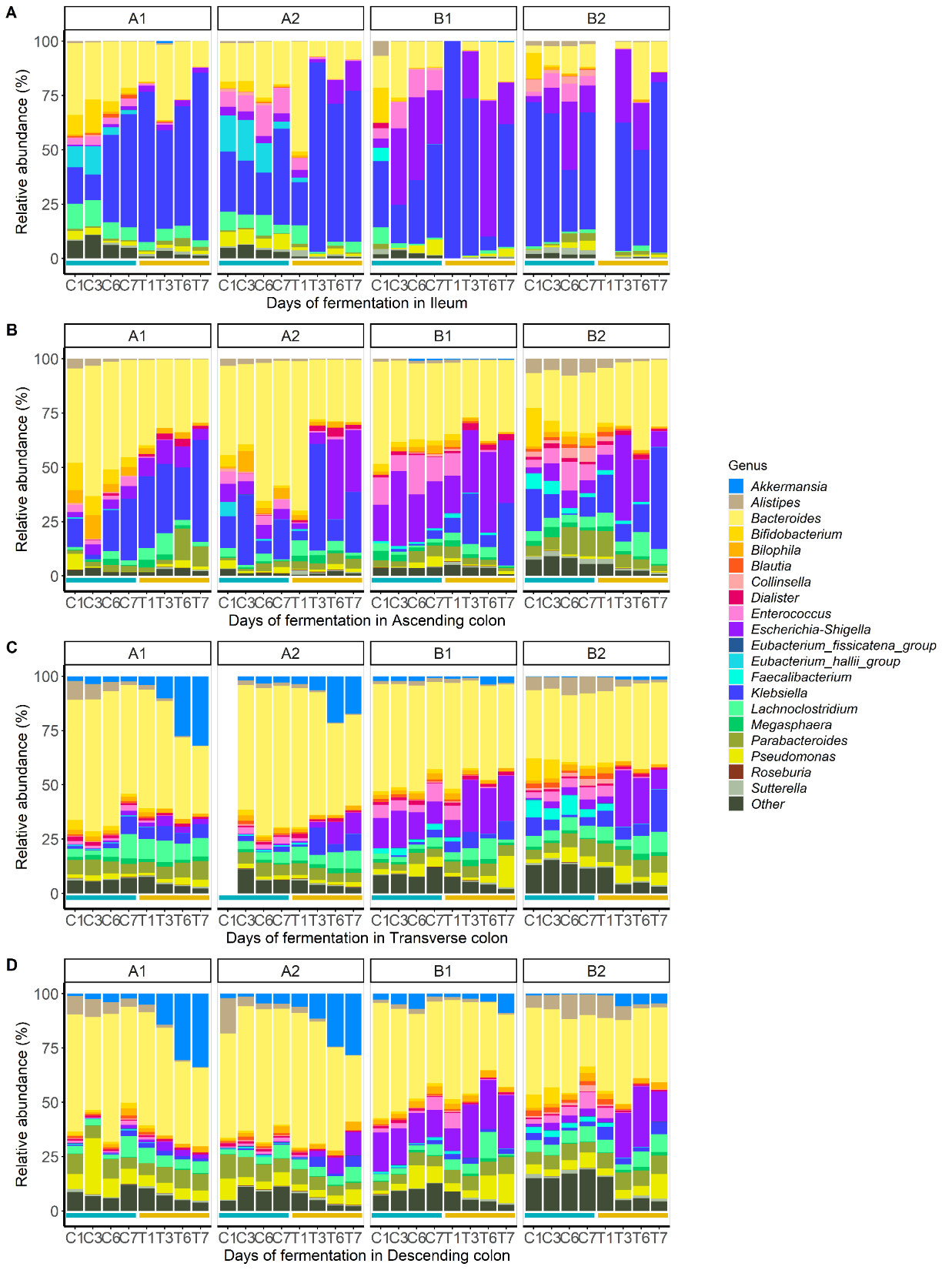


**Figure S1. Genus level relative abundance of the luminal microbiota composition following a 1-week control (C) *versus* 1-week treatment (T) across the successive gut segments of four replicates.** A1 and A2 are the duplicates from the summer microbiota while B1 and B2 are the duplicated from the winter microbiota. Control and treatment conditions are demarcated in blue and orange lines, respectively. The 20 most abundant genera are represented. Missing values are represented in white.


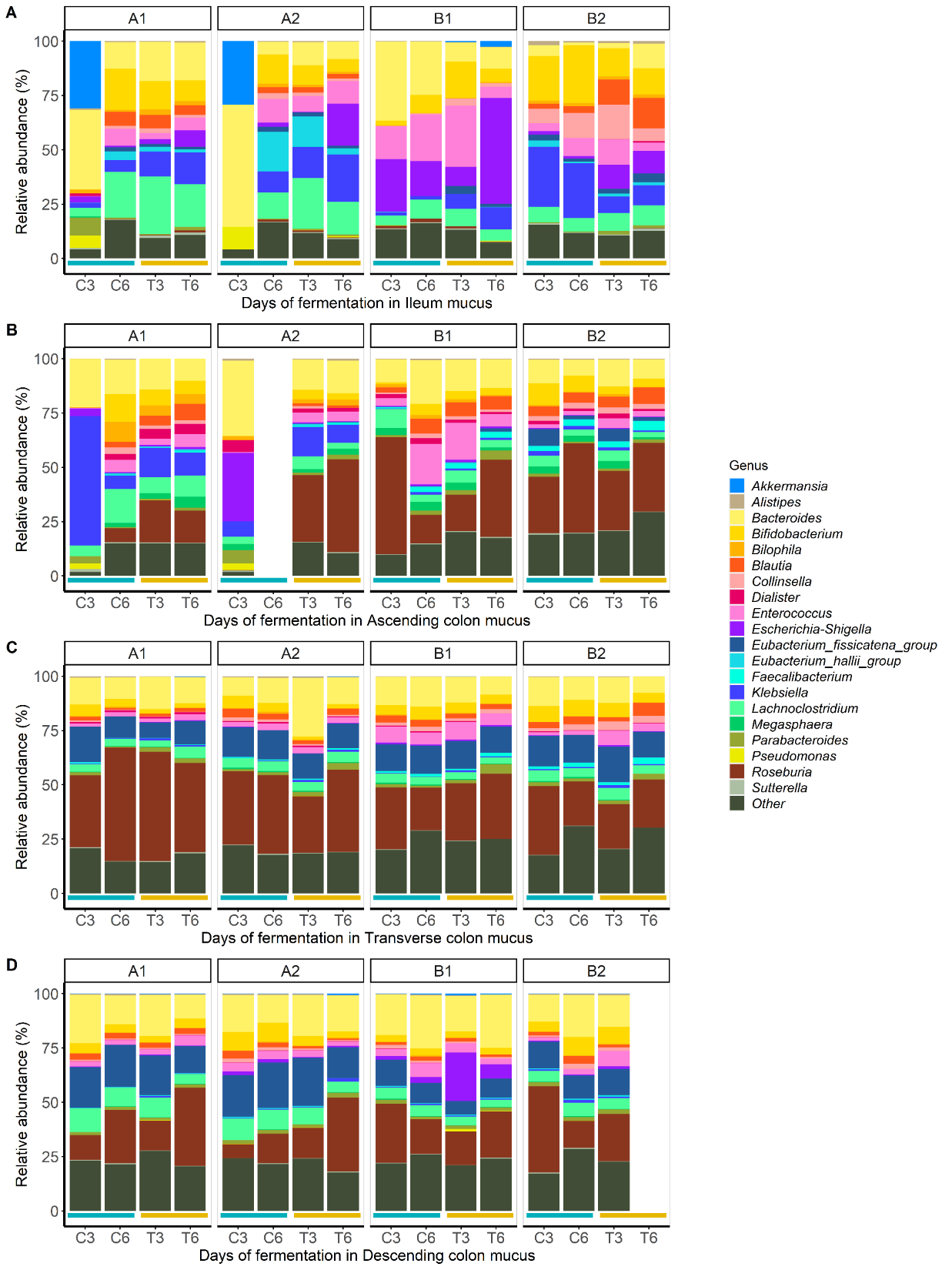


**Figure S2. Genus level relative abundance of the mucosa-associated microbiota composition following a 1-week control (C) *versus* 1-week treatment (T) across the successive gut segments of four replicates.** A1 and A2 are the duplicates from the summer microbiota while B1 and B2 are the duplicated from the winter microbiota. Control and treatment conditions are demarcated in blue and orange lines, respectively. The 20 most abundant genera are represented. Missing values are represented in white.


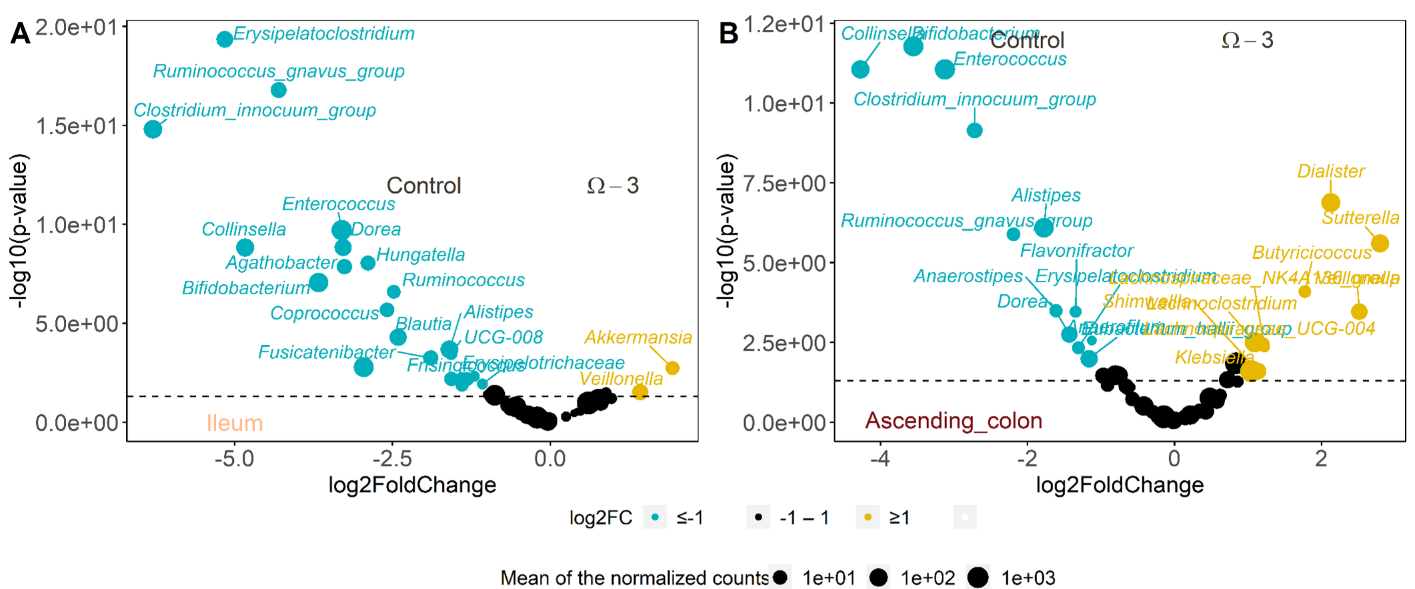


**Figure S3. Volcano plots indicating the genera significantly enriched by the omega-3 supplementation in the proximal gut segment of the luminal niche of the M-SHIME^®^.** A positive log2 fold-change indicates a stimulation of the genus under the ω-3 supplementation period (in orange) while a negative log2 fold-change indicates a decrease of the genera compared to the control period (in blue), as determined by Deseq2 analysis. The log transformed adjusted *p*-value is displayed on the y-axis and the α = .05 significance level is indicated by a dashed line.


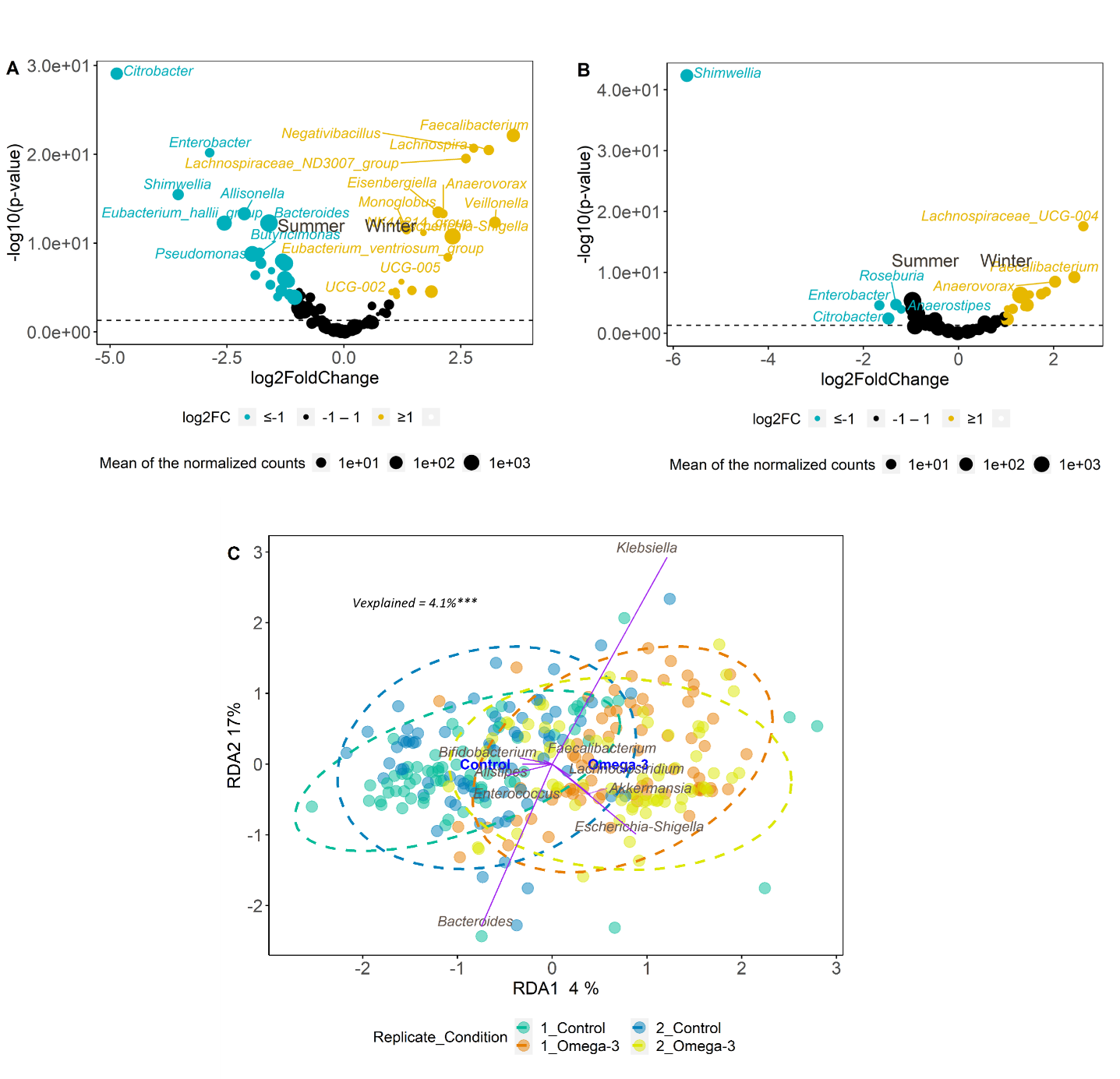


**Figure S4. Microbiota abundance and composition divergence between the summer and winter microbiota originated from the same donor in the M-SHIME^®^.** Volcano plots indicating the genera modulated during the control period **(A)** and the omega-3 supplementation **(B)** between the two microbiota seasons summer and winter. A positive log2 fold-change indicates a stimulation of the genus under the ω-3 supplementation period (in orange) while a negative log2 fold-change indicates a decrease of the genera compared to the control period (in blue), as determined by Deseq2 analysis. The log transformed adjusted *p*-value is displayed on the y-axis and the α = .05 significance level is indicated by a dashed line. **(C)** Type II scaling triplots obtained using partial distance-based redundancy analysis (db-RDA) of the microbial community composition detected using 16S rRNA gene amplicon sequencing. Factors treatment condition, and microbiota season (e.g., 1=summer and 2= winter) were set as explanatory variables (in blue) and abundances of genera as response variables (purple arrows). Only the top ten genera were displayed for adequate visibility. Axes are annotated with their contribution to the total variance. “Vexplained” indicates the variability in the gut microbiota composition explained by the variables condition, gut habitat, and lumen/mucus niches. ***indicate the *p* < .001 significance of the observed group separation, as assessed with a Permutational Multivariate Analysis of Variance (PERMANOVA) using distance matrixes.


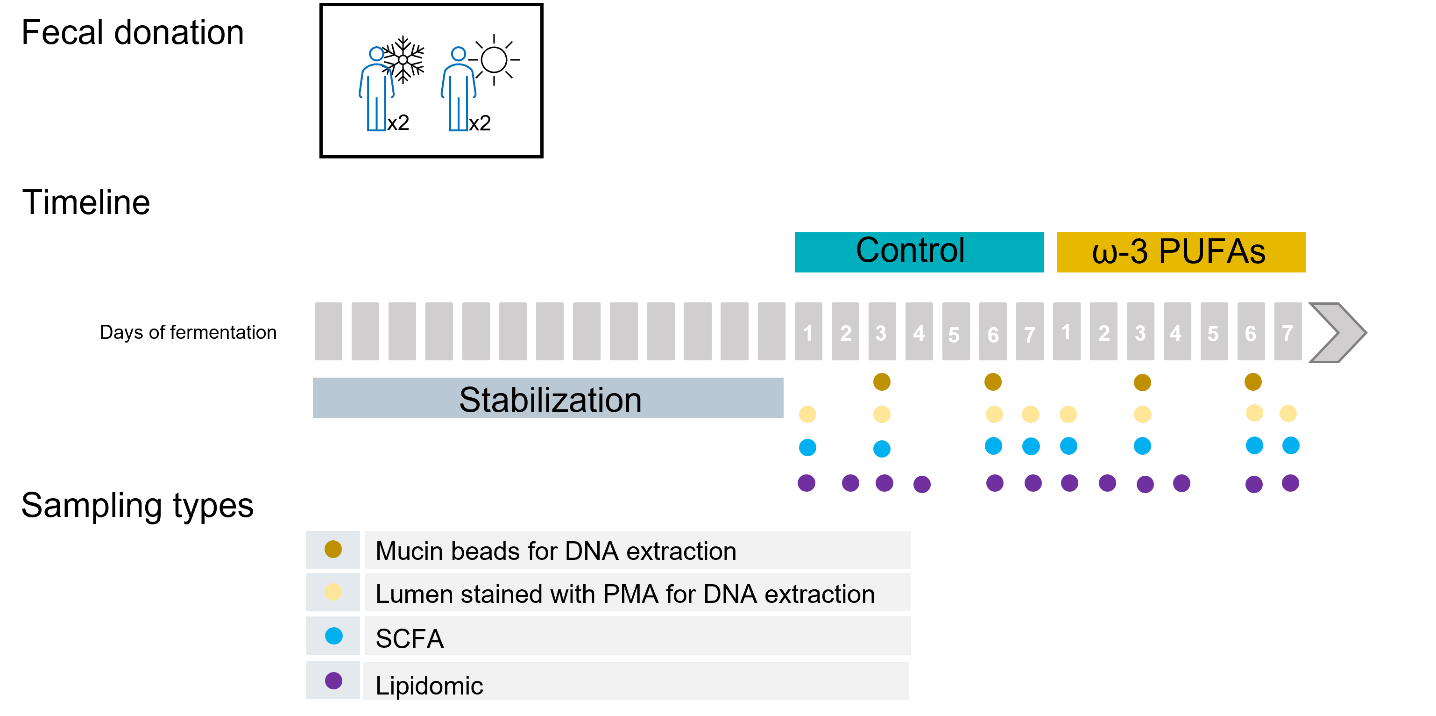


**Figure S5. Schematic view of the M-SHIME^®^ periods and sampling types**
